# GPCRs in the regulation of the functional activity of multipotent mesenchymal stromal cells

**DOI:** 10.3389/fcell.2022.953374

**Published:** 2022-08-15

**Authors:** Vadim I. Chechekhin, Konstantin Yu. Kulebyakin, Romesh I. Kokaev, Pyotr A. Tyurin-Kuzmin

**Affiliations:** ^1^ Department of Biochemistry and Molecular Medicine, Faculty of Medicine, Lomonosov Moscow State University, Moscow, Russia; ^2^ Institute of Biomedical Investigations, The Affiliate of Vladikavkaz Scientific Centre of Russian Academy of Sciences, Vladikavkaz, Russia

**Keywords:** multipotent mesenchymal stromal cells, mesenchymal stem cells, adipose tissue, hormonal regulation, beta-arrestin, heterologous sensitization, internalization

## Abstract

Adipose tissue is one of the tissues in the human body that is renewed during the whole life. Dysregulation of this process leads to conditions such as obesity, metabolic syndrome, and type 2 diabetes. The key role in maintaining the healthy state of adipose tissue is played by a specific group of postnatal stem cells called multipotent mesenchymal stromal cells (MSCs). They are both precursors for new adipocytes and key paracrine regulators of adipose tissue homeostasis. The activity of MSCs is tightly adjusted to the needs of the organism. To ensure such coordination, MSCs are put under strict regulation which is realized through a wide variety of signaling mechanisms. They control aspects of MSC activity such as proliferation, differentiation, and production of signal molecules *via* alteration of MSC sensitivity to hormonal stimuli. In this regard, MSCs use all the main mechanisms of hormonal sensitivity regulation observed in differentiated cells, but at the same time, several unique regulatory mechanisms have been found in MSCs. In the presented review, we will cover these unique mechanisms as well as specifics of common mechanisms of regulation of hormonal sensitivity in stem cells.

## Introduction

Obesity and accompanying diseases are some of the world’s most common reasons for suffering a disability or becoming unable to work. According to the World Health Organization reports, obesity has become three times more common (in the world) over the last four decades. Data from the WHO show that, in 2016, 39% of the world’s population above the age of 18 were overweight (https://www.who.int/news-room/fact-sheets/detail/obesity-and-overweight). Metabolic disorders associated with obesity, such as type 2 diabetes, atherosclerosis, arterial hypertension, ischemic heart disease, and various kinds of oncological diseases, are some of the leading causes of death and disability of the adult population in developed countries (Bjorntorp, 1997; Lee et al., 2003; Zatterale et al., 2020).

The critical factors in the development of obesity are the disruption of adipose tissue renewal mechanisms and the discoordination of synthesis, and the breakdown of storage lipids. Caused by an excess intake of nutrients, environmental factors, or genetic predisposition, it leads to the disruption of the development of new adipocytes from residential stem cells in the adipose tissue. As a result, the adipose tissue accumulates old and hypertrophied adipocytes, with the reduced sensitivity to hormonal regulation and the lack of ability to produce adipokines important for the metabolic health of the tissue. On the contrary, obesity is not always accompanied by the disorders mentioned above. In some cases of a significant increase in adipose tissue, one can observe a condition of metabolically healthy obesity (Iacobini et al., 2019). In this case, despite the increase in adipose tissue volume, it maintains its normal functioning.

Adipose tissue renewal and homeostasis rely on the postnatal stem cells of adipose tissue—multipotent mesenchymal stromal cells (MSCs) (Kalinina et al., 2011; Caplan, 2015). Firstly, they are the precursors of most newly formed adipocytes in an adult organism (Sanchez-Gurmaches and Guertin, 2014). Secondly, MSCs produce the extracellular matrix, which determines the tissue structure. Thirdly, MSCs are the key regulators of tissue metabolism through the production of paracrine and autocrine signal molecules. The MSC’s activity and the associated reparation, regeneration, and homeostasis maintenance of the adipose tissue are under strict neurohumoral regulation. This relies on a wide range of receptors on the surface of MSCs, which allow them to receive the signals from neurotransmitters and systemically acting hormones (Mendez-Ferrer et al., 2010; Sysoeva et al., 2017; Tyurin-Kuzmin et al., 2018b). In addition to that, the MSCs are also actively receptive to the signals coming from other cells of the adipose tissue, conducting constant signal crosstalk. The disruptions of hormonal regulation of MSCs and their interaction with each other and the rest of the cellular components of adipose tissue, such as adipocytes and endothelium, are the key pathogenetic elements of the development of adipose tissue diseases (Cristancho and Lazar, 2011; Vishvanath and Gupta, 2019).

## Main text

### Differentiation of MSCs is regulated by complex hormonal stimuli

In an organism, the postnatal stem cells are constantly affected by a large number of regulatory factors, hormones, and neuromediators. It is difficult to conceive that the effect of any one factor would be enough to activate the stem cell and direct it to differentiation. If that was true, one could expect that, with every increase of the concentration of the regulatory hormone, all or most stem cells would be directed to differentiation, and the pool of stem cells would be depleted in the early stages of the organism’s development. Since that is not the case, it is reasonable to suggest the existence of more complex signaling mechanisms responsible for regulating the functional activity of the MSC. And indeed, several hormones in various combinations had to be used to launch MSC differentiation in varying directions *in vitro*. For example, it is not enough to treat the cells with just the adipogenesis inductor hormone insulin to direct subcutaneous or visceral adipose tissue MSCs into adipogenic differentiation; it is also necessary to treat the cells with glucocorticoids (dexamethasone) and potentiate cAMP-dependent signaling by isobutyl methylxanthine (IBMX). In combination with cAMP-activating agents, dexamethasone opens the binding sites for cAMP-dependent transcriptional factors, which results in the expression of adipogenesis genes (Scott et al., 2011; Yang et al., 2017). The same dexamethasone is used for the osteogenic differentiation of MSCs when combined with ascorbic acid necessary for collagen production and 2-glycerolphosphate as the source of phosphate used for forming calcium phosphate mineral deposits (Langenbach and Handschel, 2013). By itself, dexamethasone activates the WNT/β-catenin signal cascade, which activates the master gene of osteogenic differentiation Runx2. In MSCs, WNT activates Frizzled receptors, members of the non-canonical GPCR family, and induces formation of the complex with transmembrane low-density lipoprotein receptor–related protein (LRP5/6) and intracellular proteins of the disheveled (DSH) family. This complex inhibits GSK3-dependent degradation of *β*-catenin. Accumulation of *β*-catenin leads to transcription activation (James, 2013) The TGFb signal factor triggers the chondrogenic differentiation of MSCs in combination with the same dexamethasone. Moreover, the TGFb factor by itself directs MSCs into the myofibroblast-like phenotype (Kim et al., 2014). Noticeably, the adipogenic differentiation is inhibited by the WNT-dependent signal pathway activated in MSCs by dexamethasone, however, in combination with other signal molecules, this glucocorticoid’s effect is reversed (Bhaskar et al., 2014). Cushing’s syndrome is a condition of increased glucocorticoid levels that leads to altered adipose tissue homeostasis. Elevated levels of glucocorticoids are associated with insulin resistance and increased gluconeogenesis. Thus, constantly elevated glucocorticoids in Cushing’s syndrome lead to diabetes mellitus development (Asensio et al., 2004).

Despite classic hormone-activated GPCRs, MSCs express adhesion GPCRs, stably associated with the extracellular matrix. Adhesion GPCRs contain a long extracellular N-terminal fragment non-covalently associated with the receptor. N-terminal fragments contain multiple adhesive motifs that bind extracellular matrix proteins. Mechanical detachment of N-terminal fragments from the receptor leads to changes in GPCR signaling, so N-terminal fragments could be described as inverse agonists. Some of these adhesion GPCRs, such as GPR116, are involved in adipogenic differentiation of MSCs (Olaniru and Persaud, 2019). Depletion of GPR116 in preadipocytes impairs adipogenic differentiation. This corresponds to a need for high density of cells seeding for adipogenic differentiation *in vitro*. MSCs also express GPCRs activated by nutrients and other non-hormonal small molecules. For example, activation of lipolysis leads to the release of free fatty acids from adipocytes. The fatty acid receptor FFAR2 inhibits adipogenic differentiation of human chorion-derived MSCs and decreases lipolysis in adipocytes (Husted et al., 2017; Iván et al., 2017). The main agonist of FFAR2 is a propionic acid that is elevated in propionic acidemia. This disease is associated with altered adipose tissue homeostasis due to blunted fatty acid oxidation and impaired lipolysis (Storgaard et al., 2020). Metabolic effects may be associated with excessive activation of FFAR2. FFAR2 antagonists have a high potential as drugs for the treatment of propionic acidemia.

Thus, MSCs are not directed to the various types of differentiation through the influence of any specific hormone or growth factor, but instead, a complex hormonal signal and local microenvironment is required for a cell to choose the direction of differentiation.

**FIGURE 1:**
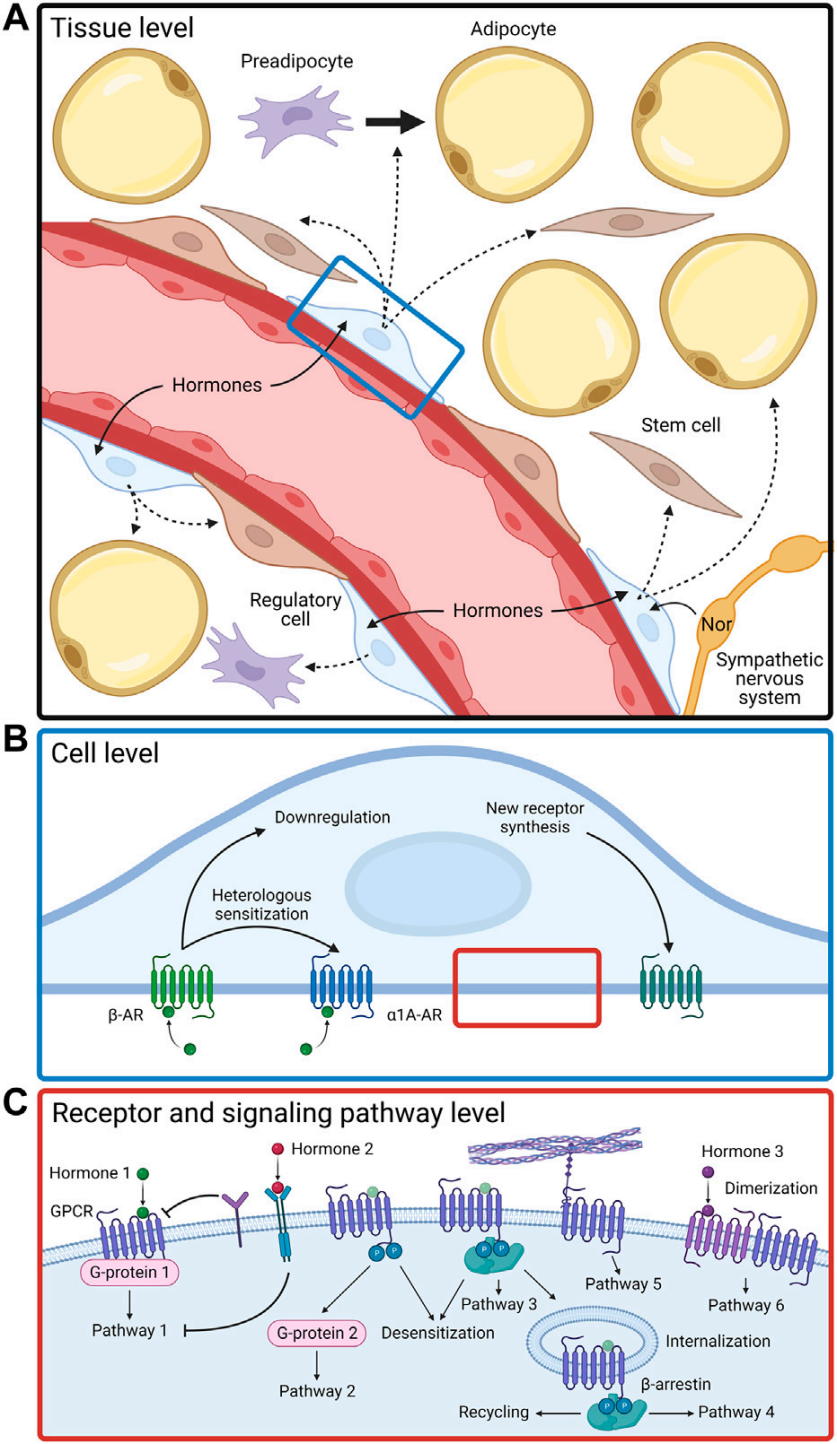
Functional activity of adipose-derived MSCs is regulated by complex mechanisms on tissue **(A)**, cellular **(B)**, and molecular **(C)** levels. Nor, norepinephrine; AR, adrenergic receptor; GPCR, G-protein coupled receptor.

The MSC’s response to a complex hormonal signal is determined by a number of factors taking effect on various levels of the functioning of these postnatal stem cells. First of all, on the molecular level, the receptors are regulated through the modification of receptors themselves as well as of elements of their signal cascades (Figure 1). For example, well-known phosphorylation of G-protein–coupled receptors (GPCRs) typically leads to the desensitization of receptors and attracts *β*-arrestin. Upon binding to the receptors, *β*-arrestin induces the internalization of the receptor, after which the receptor either recycles or degrades. Additionally, on the cellular level, for various kinds of receptors in individual cells, its content, isoform composition, and principal presence of particular receptors on the cell surface may change, which leads to a change in a given cell’s functional response to stimuli. MSCs from white adipose tissue use all the main mechanisms of hormonal sensitivity regulation observed earlier in differentiated cells, but at the same time, several unique regulatory mechanisms have been found in MSCs. Thirdly, MSCs have been able to demonstrate a new level of organization and regulation of hormonal sensitivity—the intercellular level. It is based on the presence of functional heterogeneity between cells. In recent years, evidence of an existence of a special pool of sensor cells for hormonal signals in tissues has emerged. While most tissue cells remain unreceptive to existing stimuli, these sensor cells are receptive to appearing hormones and pass on the information to other cells via paracrine stimuli, which serve the purpose of coordinating the hormonal response of the entire tissue.

### Intercellular level of regulation of MSC’s functional activity

Despite the expressed heterogeneity, the whole adipose tissue displays considerable uniformity in its response to hormones and other physiological stimuli. Thus, the adipose tissue is a uniform biological structure that functions as a single organ (Kulebyakin et al., 2020). As shown in recent years, the synchronization of functional responses of the adipose tissue is achieved through a number of mechanisms that regulate hormonal sensitivity on an intercellular level (Mendez-Ferrer et al., 2010; Schwalie et al., 2018; Ferrero et al., 2020). This group includes the mechanisms that require interaction between different functional groups of cells within a tissue.

One of the defining traits of stem cells is that, within an organism, they are located in the so-called cell niche. This term describes a particular microenvironment necessary for maintaining the viability of the stem cell as well as regulating its activity depending on the needs of the organism (Schofield, 1978). Of course, such regulation is carried out via altering the sensitivity of the stem cell to various hormonal and paracrine signals, which in the end determines whether it stays dormant, proliferates, leaves the niche, begins differentiation, etc. However, almost always, the stem cell within the niche does not possess signal and regulatory autonomy by itself and cannot respond to the signals coming from the organism (Kulebyakin et al., 2020). Therefore, of particular importance are the processes of intracellular information transmission that determine the coupling of the stem cell with a special subpopulation of cells called regulatory cells. The role of regulatory cells is to perform the initial reception of systemic organismic signals and transmit them to the stem cells located in the niche. This is the way the functional responses are synchronized, and the fine regulation of hormonal sensitivity of the stem cells occurs.

Such regulatory mechanism is clearly seen by the example of the interaction between bone marrow MSCs and hematopoietic stem cells (HSC) in the bone marrow niche. The sympathetic nervous system plays a key role in the regulation of the activity of the HSC, which is realized via the activation of β3-adrenoceptors. However, the HSCs are not innervated by sympathetic nerve fibers directly. In the bone marrow, the sympathetic nerve fibers are associated with the vessels and the perivascular MSCs, forming a so-called neuro-reticular complex (Mendez-Ferrer et al., 2010; Fielding et al., 2022). Electronic microscopy pictures show that sympathetic neurons form numerous terminals where neuromediators can be released in close proximity to the perivascular MSCs (Yamazaki and Allen, 1990). Within this system, the signals from sympathetic neurons are first transmitted to the specialized receptor MSCs, which in turn transmit these signals to the tissue-specific stem cells, regulating their activation and sensitivity to hormonal signals. The regulatory MSCs within the bone marrow can be detected through the expression of nestin (intermediate filament protein of neural progenitors). In the case of a specific depletion of the nestin-positive MSCs, the entire sympathetic regulation of the bone marrow is abolished (Mendez-Ferrer et al., 2010).

The first data supporting the existence of a similar mechanism regulating the functioning of MSCs of the white subcutaneous adipose tissue are appearing recently. Unlike the bone marrow niche, the adipose tissue lacks the interaction between the different types of stem cells. There are different kinds of functional subpopulations of MSCs within the adipose tissue. On the one hand, there are data on the presence of an activating population of MSCs that can stimulate the processes of differentiation of other cells in the adipogenic direction (Kulebyakin et al., 2019). On the other hand, a particular subpopulation of MSCs was found in the adipose tissue, which can hinder the ability of MSCs to undergo adipogenic differentiation. Such inhibiting regulatory cells were described in the MSC population within the adipose tissue of mice. A small subpopulation of cells carrying surface markers CD142 and ABCG1 was observed as part of the adipose tissue. These cells, called A-reg, were found to be able to lower the sensitivity of mouse subcutaneous and visceral adipose tissue MSCs to proadipogenic hormonal stimuli and suppress the adipogenic differentiation of preadipocytes (Schwalie et al., 2018; Ferrero et al., 2020).

The combined action of such subpopulations is necessary for normal functioning of the stem cell niche. This ensures the balance between the processes of activating the hormonal sensitivity of stem cells, stimulating their mobilization and differentiation, and long-term conservation of the stem cell in its naive state necessary for maintaining the regenerative potential of the tissue. Since the growth of adipose tissue throughout life can be mediated either by increasing the number of adipocytes (hyperplasia) or by increasing the volume of adipocytes (hypertrophy) (Muir et al., 2016), such regulatory subpopulation plays an important role in determining the manner of adipose tissue’s growth.

### Functional subpopulations of adipose-derived MSCs demonstrate various mechanisms of hormonal sensitivity regulation

Until recently, the mechanisms of intracellular regulation of hormonal sensitivity of MSCs were insufficiently explored. Nevertheless, the emergence and development of the methods of analysis of intracellular signaling at a single-cell level made a real breakthrough in this field. By now, a wide amount of data from transcriptomic analysis of the adipose tissue MSC on a single-cell level (scRNAseq) have been accumulated. Comprehensive bioinformatics analysis of this wealth of data (Ferrero et al., 2020) has shown that three distinct groups of cells differing in their functional traits and their sensitivity to regulatory signals can be distinguished. First, the stem cells of the adipose tissue possess a high level of proliferation *in vitro*, a limited sensitivity to adipogenic stimuli, and multipotency (these cells can differentiate into osteoblasts). The second group of MSCs is preadipocytes. They do not proliferate quite as well as the stem cells, can be directed to adipogenic differentiation under the influence of just insulin, and do not possess the multipotency trait. First, two groups were found in human and mouse white visceral and subcutaneous adipose tissues. The third group is the aforementioned A-reg and was found only in mouse white visceral and subcutaneous adipose tissues, but not in humans (Schwalie et al., 2018; Ferrero et al., 2020). Thus, a coordinated interaction of several types of stem cells allows the adipose tissue to function as a whole.

Two conclusions can be drawn from distinguishing the adipose tissue MSCs into functional subpopulations based on the transcriptomic analysis data. On the one hand, a suggestion can be made that there are three (mouse) or two (human) entirely different kinds of cells in the MSC population. The cells have been differentiated at some point and fixed in their phenotype. On the other hand, the exhibited separation of roles in the MSC population can be of transitory nature and labile. The data available today pointing toward the second hypothesis are correct. Several experiments have been able to produce populations made up of the descendants of a single MSC. A functional heterogeneity similar to that of the parent population was observed in these groups of cells (Ylöstalo et al., 2008; Rennerfeldt and Van Vliet, 2016).

Other data supporting this hypothesis were obtained in our laboratory. We have examined the sensitivity of MSCs to hormones activating the cAMP-dependent signal pathway using a genetically encoded biosensor that can register the activation of protein kinase A (PKA) on the single-cell level (Zhang et al., 2018). We have found that subcutaneous white adipose tissue MSCs exhibit heterogeneity on activating cAMP-dependent signal cascades in response to hormones. Only about 40% of the population’s cells are able to respond to the stimulation. These cells respond to various hormones without strong specificity, and a single individual cell can form a response to several different hormones. The second half of the cells of the population respond to none of the hormones. The addition of non-specific adenylyl-cyclase activator forskolin does not lead to a substantial increase in the number of responsive cells. In order to verify whether MSCs can transit between a responsive and a non-responsive group of cells, we have obtained clones of MSCs from single cells. The clones were produced from both the hormone-responsive and the hormone non-responsive cells. It turned out that, after only 2 weeks of culture, the heterogeneity on responsiveness to hormones and forskolin is formed again—some cells were able to form a response and some were not (Tyurin-Kuzmin et al., 2020b). To find out on which level the responsiveness of several MSCs to cAMP-activating hormones is disrupted, we have incubated cells with a non-degradable analog to cAMP 6-Bnz-cAMP. This molecule activates the biosensor’s response in all cells of the population. These results can be interpreted such that adenylyl-cyclase does not function or is not expressed in the cells that are non-responsive to forskolin but responsive to 6-Bnz-cAMP. Since ten isoforms of adenylyl-cyclase have been shown to exist in mammals’ cells, their simultaneous expression, or the lack thereof in a single cell, is rather hard to test. We have analyzed the expression of adenylyl-cyclase MSCs with scRNAseq. According to the data from bioinformatics processing, over half the cells in the population of subcutaneous white adipose tissue MSCs do not express mRNA of either of the ten isoforms of adenylyl-cyclase (Tyurin-Kuzmin et al., 2020b). Thus, MSCs can regulate their responsiveness to hormones by selective and regulated expression of the participant molecules of signal cascades.

### Receptor level of regulation of MSCs’ responsiveness to hormones

Suppressing the receptiveness of MSCs to hormones can occur not only on the level of receptor-associated signal cascades but also by regulating the accessibility of receptors themselves. Accordingly, classical ways of desensitization and down-regulation of the seven-domain receptors by phosphorylation, association with *β*-arrestin, and subsequent internalization are widely used by MSCs. At the same time, different receptors on the surface of MSCs require considerably different times for induction of internalization. We have demonstrated that when subcutaneous white adipose tissue MSCs are treated by angiotensin II, an extremely fast internalization of angiotensin receptor AT1R occurs right after the activation of calcium signaling (Sysoeva et al., 2017). Registering the hormonal responses on the single-cell level has shown that the initial addition of a hormone leads to activating calcium signaling in over 50% of the cells in the population. However, only 2–5% of the cells in the population are able to respond to the second addition of angiotensin II. This is due to the fact that AT1R is internalized immediately after binding the ligand, in 30 s–1 min (Sysoeva et al., 2017). Furthermore, the antibodies to this receptor also can act as the inductor of internalization of the receptor (Ageeva et al., 2018). *β*-Adrenoceptors are also subject to internalization in MSCs in case of excessive action of noradrenaline. This process, however, occurs over the course of an hour (Tyurin-Kuzmin et al., 2016). *α*1- and *α*2-adrenoceptors as well as purinergic receptors in MSCs are weakly desensitized for several hours since the repeated stimulation of individual cells with noradrenaline or adrenoceptor agonists leads to reproducible calcium responses over the course of several hours (Kotova et al., 2014; Kotova et al., 2018). It should be noted that aforementioned class A GPCRs show similar internalization kinetics in case of exogenous expressing: AT1R is internalized after 5 min (Fessart et al., 2005) or 20–30 min (Cao et al., 2020) of hormone action and *β*-adrenoceptors after 10–20 min (Boursier et al., 2020). The difference in the kinetics of internalization of these receptors in MSCs can be explained by the presence of regulatory machinery, which includes specific adaptor and scaffold proteins, the exact mechanisms of which have to be studied.

Another important feature of MSCs is that the ability to internalize the receptors can vary between different groups of MSCs. We have isolated a small subpopulation of MSCs, characterized by its expression of AT1R as well as co-expression of other isoforms of angiotensin receptors, such as AT2R and Mas (Ageeva et al., 2017; Sysoeva et al., 2017). In these cells, the internalization of AT1R is fully suppressed, which may be a consequence of its heterodimerization with other angiotensin receptors on the surface of the cell (AbdAlla et al., 2001; Tyurin-Kuzmin et al., 2020a). The inability to internalize AT1R significantly alters the functional characteristics of this small subpopulation of MSCs that exhibits an increased adipogenic potential (Sysoeva et al., 2017).

Heterodimerization may also inhibit GPCR activation. One of the well-studied examples of such action is Hedgehog-dependent inhibition of MSC adipogenic differentiation. Smoothened is a Frizzled family GPCR, which has not its own ligand and is active in this ligand-absent state. Heterodimerization of Smoothened with another membrane receptor Patched leads to inhibition of Smoothened. Binding of Patched receptor to its ligand Hedgehog leads to separation of Patched from Smoothened and subsequent activation of the latter (James, 2013).

Internalization of receptors does not always lead to desensitization. As such, in several cases, internalization of the receptor is a prerequisite for increasing its activity. For example, it is shown that, for the parathyroid hormone receptor (PTHR), internalization of the hormone–receptor complex leads to a significant increase in the amplitude and length of cAMP production (Ferrandon et al., 2009). In the classical case, long-term stimulation of the G-protein–coupled receptor leads to its phosphorylation and *β*-arrestin–dependent desensitization due to the disturbance of interaction between the G-protein and the receptor (Luttrell and Lefkowitz, 2002). In the case of the type 1 PTHR, the Gs-proteins keep their interaction with the receptor even after the association of *β*-arrestin and the internalization of the hormone–reception complex. If PTH analogs that are irreversibly bound to the receptor are used, then a long-term cAMP-dependent signal is observed only if the receptor is internalized. Inhibition of internalization leads to forming a transitionary response even in the presence of the hormone bound to the receptor (Ferrandon et al., 2009). Furthermore, overexpression of *β*-arrestin in the case of *β*-adrenoceptors leads to the faster termination of the cAMP-dependent signal, while in the case of PTHR1, it leads to an increase of cAMP-dependent signaling (Feinstein et al., 2011).

GPCR density at the cell surface may also be a factor of switching between different signaling cascades. For example, the amplitude of PTH1R-dependent activation of AC/cAMP signaling does not depend on the density of receptors on the cell surface. But, besides G_s_, PTH1R may activate G_q_ and subsequent PLC/Ca^2+^ signaling, and PLC activity is dependent on the number of receptors on the cell surface (Guo et al., 1995; Takasu et al., 1999). Experiments with exogenous expression of different amounts of receptors in the PTH1R-null cells demonstrated that, in the case of expression level of 20,000–100,000 receptors per cell, PTH induces the maximal level of cAMP production, but PLC is not activated. The increase of PTH1R level up to 800,000 receptors per cell leads to dose-dependent up-regulation of PLC in response to PTH. Thus, at a high level of PTH1R expression, G_q_/PLC/Ca^2+^-dependent signaling may begin to dominate over the G_s_/AC/cAMP pathway. It can be assumed that the same mechanism of signaling switching can be realized in the case of local redistribution or oligomerization of PTH1R molecules.

PTH is the main regulator of calcium homeostasis, and its main targets are bones and kidneys. Nevertheless, disruptions of the receptiveness of adipose tissue MSCs to PTH lead to the abnormal differentiation of these cells—the development of areas of ectopic ossification in adipose tissue (Pignolo et al., 2011; Vorontsova et al., 2021).

Dysregulation of adipose tissue homeostasis in obesity is often associated with desensitization of *β*3-adrenergic receptors. The proinflammatory state in adipose tissue suppresses *β*3-adrenoceptors, which in turn decreases lipolysis. Impaired lipolysis augments lipid accumulation in adipose tissue and leads to pathological positive feedback in obesity (Valentine et al., 2022).

### Switching the receptor-activated signaling cascades

The functional activity of stem and regulatory cells of the adipose tissue is regulated not only through selective turning of hormone receptors on and off but also through switching the signaling cascades activated by the hormone. There are several variants where one hormone affecting various cells containing the same set of surface receptors can activate different functional cell responses.

For the first example, we can look at the alteration of the connection of the receptor to one or another signal cascade as a result of post-translational modifications of the receptor. Phosphorylation of seven-domain receptors is historically considered to be associated with fast desensitization. Nevertheless, several mechanisms associated with the phosphorylation of these receptors leading not to suppression but to switching of the way of signal transduction have been demonstrated. Normally, *β*-adrenoceptors are associated with the trimeric Gs-protein and activate the signal cascade of adenylyl-cyclase/cAMP/PKA. However, it was shown in a number of cells that excessive stimulation of *β*-adrenoceptors and long-term activation of PKA lead to the phosphorylation of the receptor. Phosphorylated *β*-adrenoceptors exhibit increased affinity to the trimeric Gi-protein (Daaka et al., 1997), which leads to the inhibition of cAMP-dependent signal cascade, the opposite effect to the regular cellular response to the stimulation of *β*-adrenoceptors. Even though these mechanisms were at first demonstrated on cardiomyocytes, recent data point to a possibility for the realization of such mechanism in the adipose tissue as well (Hajifathali et al., 2014). Another example is the PTH1R receptor which can normally activate both Gs and Gq proteins. PKA-dependent phosphorylation of seven serine residues on the C-terminal tail of PTHR1 leads to a dramatic decrease in Gq/PLC-dependent signaling (Tawfeek and Abou-Samra, 2008).

For the second example of the switching of the receptor-associated signal cascade, we can look at the participation of adapter proteins in transmitting the signal from a number of regulatory receptors of MSCs, for example, PTHR1, which normally couples with the trimeric Gs-protein and activates cAMP-dependent signaling. In the case of interaction of the receptor with the adapter protein NHERF (Na+/H+-exchange regulatory cofactor), intracellular signaling activated by this receptor changes. NHERF increases the receptor’s affinity for the trimeric Gq-protein, which induces phosphoinositide-dependent and calcium signaling. Moreover, the NHERF2 isoform can also activate the Gi-protein, which additionally inhibits adenylyl-cyclase and cAMP-dependent signaling (Wang et al., 2010; Broadbent et al., 2017). Age-associated changes in NHERF-dependent mechanisms of regulation of intracellular signaling from PTHR1 may be associated with the development of osteoporosis (van Gastel et al., 2021). Thus, the adapter proteins associated with the receptors can switch intracellular signaling activated in different cells by the same hormone and its receptor.

**FIGURE 2:**
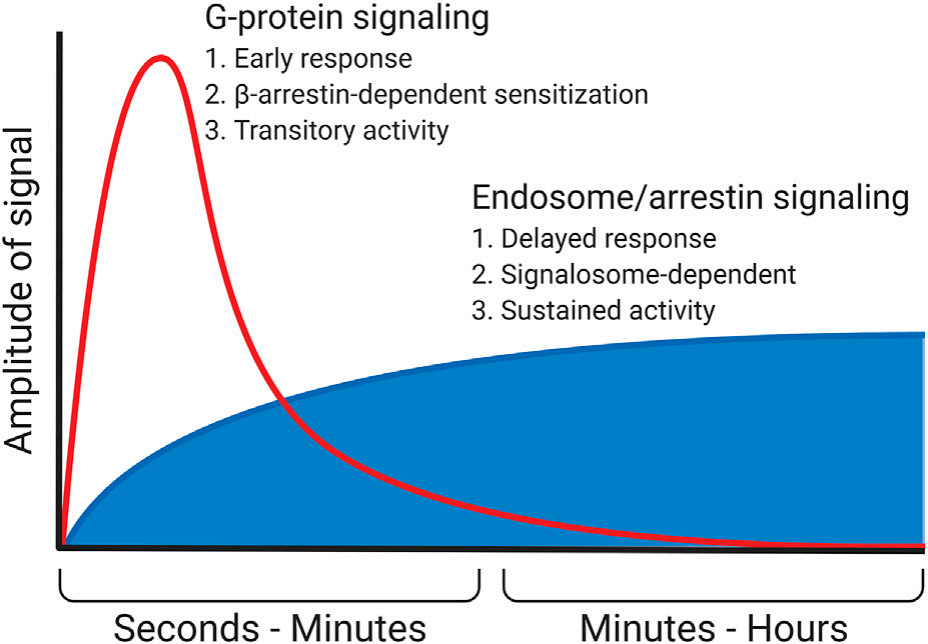
Signaling in endosomes. In particular, the *β*-arrestin–dependent one is started after the desensitization of the receptor and the termination of the G-protein–dependent signal. This signal most often possesses a lower amplitude but is more prolonged. In some cases (e.g., PTHR), *β*-arrestin does not displace G-protein but instead stabilizes its complex with the receptor, enhancing and prolonging the G-protein–dependent signal.

*β*-Arrestin serves as the third example of switching intracellular signaling between different signal cascades. Aside from mediating the desensitization of the receptor and inducing its internalization, *β*-arrestin itself is a scaffold protein. It organizes components of signal cascades that are not considered canonical for GPCRs. For example, *β*-arrestin binds Raf-1 protein kinase and other components of the МАР-kinase signal cascade, activating them. As a result, the signaling is switched from the typical for seven-transmembrane receptors calcium-dependent and cAMP-dependent signal cascades to the МАР-kinase one, which is more characteristic of the growth factor receptors (Luttrell et al., 2001; Shenoy and Lefkowitz, 2011). In addition, *β*-arrestin can bind and activate a number of other signal molecules, such as Src kinase, some isoforms of phosphodiesterase, and phospho-inositol-3-kinase (PI3K) (Perry et al., 2002; Peterson and Luttrell, 2017). Which signal cascade will be activated by a specific receptor–β-arrestin complex depends on at least two factors. The first is other participants of the signaling complex assembled on the receptor. These adapter proteins, interacting with *β*-arrestin, change its conformation and, accordingly, its affinity for various proteins. The second is post-translational modifications of the receptor and *β*-arrestin itself. Multiple modifications of the receptor and a scaffold protein, including phosphorylation at multiple sites and different patterns of ubiquitination, promote various downstream signaling molecules (Smith and Rajagopal, 2016). More data are now accumulating that senescence and the development of age-associated diseases change the spectrum of predominantly activated *β*-arrestin–dependent signaling cascades. For example, glucagon-like peptide 1 (GLP1), which is an important regulator of nutrient homeostasis, regulates the balance between adipogenic and osteogenic differentiation of MSCs in a *β*-arrestin/Erk-dependent manner (Lee et al., 2015). Age-related changes in the regulation of *β*-arrestin–dependent internalization of GLP1 receptor may play a role in the development of type II diabetes (van Gastel et al., 2021). Intracellular signaling activated on endosomes by *β*-arrestin usually has a lower amplitude and longer duration than G-protein–dependent signals from the receptor (Figure 2) (Luttrell and Gesty-Palmer, 2010).

The alteration of the expression of *β*-arrestin plays an important role in the regulation of hormonal sensitivity of preadipocytes and adipocytes. For example, for 3T3-L1 preadipocytes, it was shown that insulin stimulation causes a suppression of *β*-arrestin expression and the associated shutdown of the internalization of β2-adrenoceptors (Hupfeld et al., 2003). The result is a hypersensitization of β2-adrenergic receptors and cAMP-dependent signaling, which is necessary for the induction of the initial stages of adipogenic differentiation (Jia et al., 2012). Interestingly, in mature adipocytes, the crosstalk between insulin signaling and *β*-adrenoceptors completely changes its direction. Excessive action of insulin on adipocytes leads to specific uncoupling of *β*-adrenergic receptors from protein kinase A due to inhibition of the A-kinase–associated proteins (AKAPs) responsible for this. Consequently, in mature adipocytes, insulin heterologically decreases the sensitivity of *β*-adrenergic receptors (Zhang et al., 2005). Thus, the availability of *β*-arrestin and its interaction partners in a particular cell can significantly alter the signaling cascades and the functional responses activated by the ligand binding to its receptor.

*β*-Arrestin also serves as a modulator of glucocorticoid receptor signaling. Down-regulation of *β*-arrestin-1 leads to glucocorticoid receptor–dependent expression of E3 ubiquitin ligase. The latter targets the intracellular receptor which leads to its degradation (Petrillo et al., 2019). In turn, dexamethasone induces up-regulation of *β*-arrestin-1 accompanied by down-regulation of *β*-arrestin-2, which leads to redirection of GPCR-dependent signaling from G-protein–dependent Ca^2+^ activation to *β*-arrestin–dependent activation of Erk1/2 (Oakley et al., 2012). Thus, *β*-arrestin may be the point of intersection of intracellular signaling of GPCRs and glucocorticoid receptors.

**FIGURE 3:**
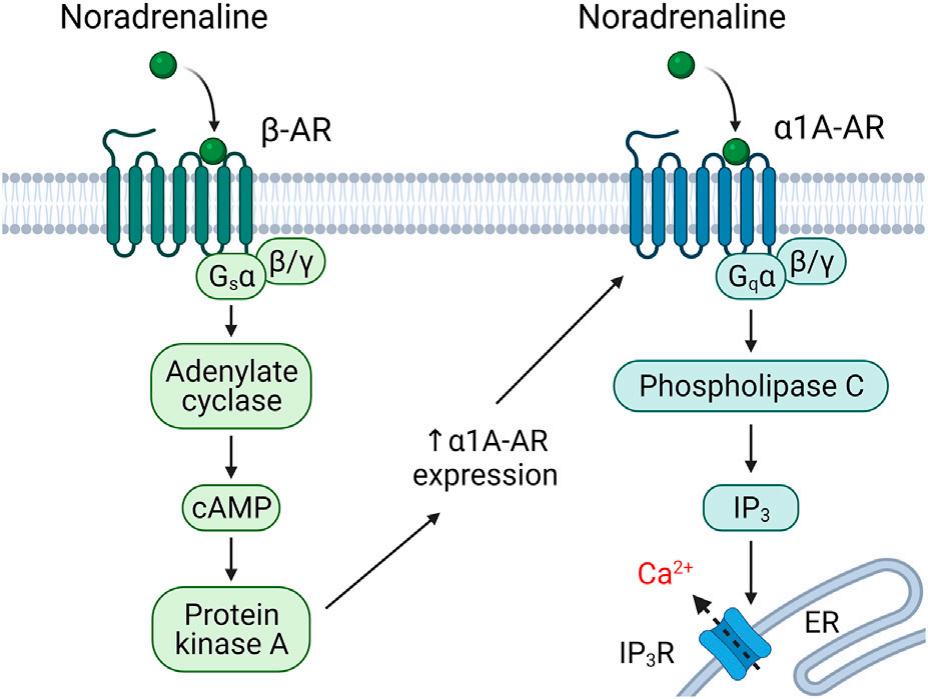
Heterologous sensitization of alpha1A-adrenergic (*α*1A-AR) receptors. Stimulation of adipose-derived MSCs with noradrenaline leads to beta-adrenergic (*β*-AR)-dependent sensitization of alpha1A-adrenoceptors (Tyurin-Kuzmin et al., 2016).

The fourth mechanism of the multidirectional effects of hormones on different cells is the varying isoform composition of the receptors exhibited on the surface of various cells. Noradrenaline, for example, can activate a group of α1-adrenoceptors, which activate phosphoinositide/calcium-dependent signaling, and *β*-adrenoceptors that activate and α2-adrenoceptors that inhibit cAMP-dependent signaling *via* Gs and Gi trimeric proteins, respectively. Recently, we described in the subcutaneous white adipose tissue MSCs a mechanism of rapid transient change of the isoform pattern of adrenergic receptors presented on the surface of cells (Figure 3) (Tyurin-Kuzmin et al., 2016). This mechanism called heterologous sensitization is not seen in other postnatal cells. Normally, noradrenaline affecting the MSCs is sensed primarily with *β*-adrenergic receptors. cAMP-dependent signaling started by them leads, on the one hand, to desensitization of *β*-adrenergic receptors and, on the other hand, to delayed *α*1A-adrenoceptor expression on the surface of cells. Firstly, this results in that the cells respond to repeated exposure to noradrenaline with a phosphoinositide/calcium-dependent signal cascade, rather than a cAMP-dependent one; secondly, the responsiveness of MSCs to noradrenaline increases more than five times (Tyurin-Kuzmin et al., 2016; Tyurin-Kuzmin et al., 2018b). The phenomenon of heterological sensitization is transient; 24 hours later, the cell responsiveness returns to original values. This phenomenon is specific to the young primary MSCs. With replicative aging of stem cells or their immortalization, heterological sensitization disappears (Tyurin-Kuzmin et al., 2018a). Thus, heterologous sensitization of MSCs is a complex mechanism for the regulation of hormonal sensitivity, which includes simultaneous down-regulation of some receptors and sensitization of others and, as a result, switching the signaling activated by noradrenaline in these cells.

## Conclusion

The regulation of the functional activity of multipotent mesenchymal stem cells of adipose tissue is crucial for their coordinated action during the processes of reparation and regeneration of tissue and maintaining its homeostasis. A wide variety of mechanisms and forms of regulation of MSC hormonal sensitivity ensure a harmonic interaction between organisms, adipose tissue, and MSCs. As a result, the MSC function is finely tuned to the dynamic needs of the tissue. Adipose tissue stem cells had to maintain heterogeneity to perform their functions. It is provided, among other things, by the distinction between individual cells in the sensitivity to hormones, which is tightly regulated. In addition to the mechanisms of hormonal sensitivity regulation usual for most differentiated cells, unique forms of regulation can be found in stem cells. For example, the phenomenon of heterologous sensitization of adrenergic receptors is known during embryonic development, but it is infrequent in an adult organism.

Furthermore, a significant part of MSCs show a reversible decrease or even a complete loss of sensitivity to hormones. This trait is dictated by the specifics of MSC functioning—the need to maintain a certain pool of stem cells in an undifferentiated state as a regenerative potential of adipose tissue. The various regulatory mechanisms for controlling cellular sensitivity to hormones require an integrated response mediated by a group of regulatory MSCs. Understanding the mechanisms of regulation of stem cells’ hormonal sensitivity is especially important in the light of the development of a new direction in biomedical science—regenerative medicine. One of the critical tasks of regenerative medicine is the treatment of diseases by controlling the body’s regenerative processes. A detailed elucidation of the molecular mechanisms of regulation of cells’ functional activity and the mechanisms of switching hormonal sensitivity will make it possible to control endogenous regenerative processes in a predictable manner.
